# Rethinking symbolic and visual context in Referring Expression Generation

**DOI:** 10.3389/frai.2023.1067125

**Published:** 2023-03-21

**Authors:** Simeon Schüz, Albert Gatt, Sina Zarrieß

**Affiliations:** ^1^Faculty of Linguistics and Literary Studies, Bielefeld University, Bielefeld, Germany; ^2^Natural Language Processing Group, Department of Information and Computing Sciences, Utrecht University, Utrecht, Netherlands

**Keywords:** Referring Expression Generation (REG), visual context, Natural Language Generation, scene context, Vision and Language, language grounding

## Abstract

Situational context is crucial for linguistic reference to visible objects, since the same description can refer unambiguously to an object in one context but be ambiguous or misleading in others. This also applies to Referring Expression Generation (*REG*), where the production of identifying descriptions is always dependent on a given context. Research in REG has long represented visual domains through *symbolic* information about objects and their properties, to determine identifying sets of target features during content determination. In recent years, research in *visual REG* has turned to neural modeling and recasted the REG task as an inherently multimodal problem, looking at more natural settings such as generating descriptions for objects in photographs. Characterizing the precise ways in which context influences generation is challenging in both paradigms, as context is notoriously lacking precise definitions and categorization. In multimodal settings, however, these problems are further exacerbated by the increased complexity and low-level representation of perceptual inputs. The main goal of this article is to provide a systematic review of the types and functions of visual context across various approaches to REG so far and to argue for integrating and extending different perspectives on visual context that currently co-exist in research on REG. By analyzing the ways in which symbolic REG integrates context in rule-based approaches, we derive a set of categories of contextual integration, including the distinction between *positive* and *negative semantic forces* exerted by context during reference generation. Using this as a framework, we show that so far existing work in visual REG has considered only some of the ways in which visual context can facilitate end-to-end reference generation. Connecting with preceding research in related areas, as possible directions for future research, we highlight some additional ways in which contextual integration can be incorporated into REG and other multimodal generation tasks.

## 1. Introduction

In the most natural forms of conversation, speakers are situated in a shared environment and, while talking, perceive the visual world around them. In such a scenario, speakers commonly produce utterances **about** particular visually perceivable objects, and they do so **within** a rich visual context that contains far more objects than the ones being mentioned explicitly. A prime example of this kind of language use are referring expressions, i.e., linguistic expressions that refer to visible objects located in the environment. When referring, speakers need to describe objects in a way that an interlocutor is able to identify the target among other objects in the visual context. Hence, the words they produce are grounded in visible aspects of the target object being described, but the particular choice of words will also depend on the visual context around them. For example, “the dog” is sufficiently informative in many cases, but requires further specification if multiple dogs are visible.

In recent years, research at the intersection of Natural Language Processing and Computer Vision, i.e., in the area of Language & Vision (L&V), has made important steps toward modeling situated interaction by building multi-modal neural language models that ground linguistic representations in visual inputs (i.e., images). State-of-the-art L&V models have been shown to achieve impressive performance in generating or understanding utterances **about** visual objects, actions, and relations in visual scenes (cf. Mogadala et al. 2021). Yet, their ability to model communication **within** visual context remains elusive, as existing L&V tasks and datasets rarely offer any explicit ways of manipulating the non-linguistic (or *situational*, Meibauer 2012) context. *Referring Expression Generation (REG)*, a well-known task in Natural Language Generation (Krahmer and van Deemter, 2019), is one of the few areas where the modeling of situational context has been viewed as central: an REG system's task is to generate a description for a given object, which would allow a hearer to identify the intended referent in the given situation (Reiter and Dale, 2000). The focus on *identification* as a testable success criterion frames reference as a self-contained task, which makes it an attractive research subject for investigating the otherwise elusive effects of situational context on language generation.

Research on REG has been conducted in many different set-ups and paradigms, with different underlying aspects and notions of context which, to date, have not been systematically compared or integrated. In this review, we mainly contrast between *symbolic* and *visually grounded* (henceforth *visual*) approaches in REG, which both follow the underlying task formulation introduced above but differ fundamentally in their inputs. In symbolic REG, visual environments are commonly regarded as the primary use case (e.g., Dale and Reiter 1995). However, symbolic REG assumes that the generation process starts *after* the categorization of the scene and the objects in the visual context, and that perceptual features have been bundled into symbolic properties. As these properties are entirely decoupled from the perceptual sensations to which they refer, this kind of information representation can be regarded an example of Harnad (1990)'s *Symbol Grounding Problem*. In contrast to this, approaches in visual REG directly operate on low-level visual representations of e.g., objects in photographs, mostly using neural generation models. Importantly, in both cases, the result of the generation process is sequences of words (i.e., symbols) – hence, in visual approaches, the perceptual input has to be mapped to words and sentences (which, in turn, are visually grounded).

In this work, as an initial step toward a deeper understanding of visual context in multimodal generation tasks, we will characterize some of the different ways in which context is utilized to achieve pragmatic goals in reference, and thus affects the generation of referring expressions in natural images. Our main goal is to provide a systematic review of the types and functions of visual context across various approaches to REG so far and to argue for integrating and extending different perspectives on visual context that currently co-exist in research on REG. In contrast to previous surveys on this task (Krahmer and van Deemter, 2012, 2019), we consider different modeling paradigms, i.e., rule-based approaches for symbolic inputs as well as visual REG with neural generation models, and pay particular attention to the various ways in which the situational context can affect expression generation. To this end, we leverage the opposing characteristics of different REG paradigms by analyzing the ways in which context is used in symbolic approaches, and using those insights as a guidance for investigating possible functions of visual context in neural REG. More precisely, we argue that different lines of research in symbolic REG can be re-framed as including symbolic context in additional ways and on different stages of processing, in order to satisfy different pragmatic objectives. We analyze those approaches in terms of the various ways in which context objects affect the content of generated expressions, and derive from this a distinction between different *categories of contextual integration*. After this, we turn our attention to context in visual REG, and explore whether corresponding functions of context are reflected in existing REG models. Finally, we highlight directions for future research regarding the types of context not reflected in the visual REG literature so far.

While this article focuses on REG, the issues discussed here are not confined to this specific task. First, the opposing characteristics of symbolic and visual REG reflect more general patterns of multimodal language processing: Whereas symbolic approaches offer crisp task definitions and transparent processing stages, they are hard to apply to more natural settings. Visual approaches, on the other hand, are compatible with raw visual inputs, but at the expense of less overt ways of processing. Second, the question of how visual context affects language generation and processing is crucial for a variety of Vision and Language (V&L) tasks involving linguistic references to visible objects, such as Image Captioning or Visual Question Answering, and ultimately, multimodal language processing in general.

## 2. The REG task

In Referring Expression Generation (REG), the goal is to generate descriptions for entities, which allow their identification in a given context (Reiter and Dale, 2000). As mentioned in the previous section, this task has undergone notable change over the past decades: Pioneering work focused on prototypical forms of reference and represented the communicative situations for referential acts through high-level symbolic information about individual objects, abstracting away from e.g., visual representations (Section 2.1.1). This core formulation of the task was gradually extended in subsequent work, with the aim of achieving complete algorithms that can capture linguistic variation and domain-specific requirements (Section 2.1.2). Recent work in REG has shifted to more natural settings such as objects in natural images, enabled by the capability of neural modeling to process low-level perceptual information and the availability of large-scale vision and language corpora such as RefCOCO (Kazemzadeh et al. 2014; Section 2.2). In this section, we look at these lines of research in REG and highlight general differences between symbolic and visual approaches.

Importantly, in natural conversation, a variety of linguistic as well as non-linguistic devices can be used for referential actions. In this article, in line with most work in REG, we focus on the *one-shot* generation of referential noun phrases, leaving aside, for example, deictic gestures and reference via proper nouns or pronouns. Extending this view, research on *multimodal* REG (van der Sluis and Krahmer, 2001; Krahmer and van der Sluis, 2003; Kranstedt and Wachsmuth, 2005; Kranstedt et al., 2006; Piwek, 2009) includes pointing gestures as complementary devices for referring to visible objects. Going beyond one-shot reference, Zarrieß and Schlangen 2016 generate incrementally produced *installments* to gradually guide the addressee to the intended referent. In Fang et al. (2014, 2015), both installments and deictic gestures are used to account for perceptual mismatches between humans and artificial agents in situated dialog. Mental states and perceptual capabilities of interlocutors play an important role in natural communication, but are rarely considered in REG (but see e.g., Horacek 2005 for an exception).

### 2.1. Symbolic REG

#### 2.1.1. The core formulation

Generating references to objects has been a long-standing field of interest in computational linguistics (e.g., Winograd 1972; Appelt 1985; Appelt and Kronfeld 1987; Kronfeld 1989). Whereas earlier works considered a variety of pragmatic goals, influential works from the 1990s focused on the problem of *identification* (Dale, 1989, 1992; Reiter, 1990; Dale and Reiter, 1995). We refer to this line of work as the *core formulation* for REG. With identification as the sole communicative aim, REG algorithms can be considered successful iff they generate *distinguishing descriptions*, which apply to the target but not to any other entity in the given domain (in ways that are clear to the listener, Reiter and Dale 1992, and given that such descriptions exist for the given situation). In more detail, a referring expression has to take account of the state of the hearer's knowledge (coined the *principle of sensitivity*) and provide sufficient information to identify the intended referent (*adequacy*), without being overly informative (*efficiency*, Dale and Haddock 1991a). This builds on a Gricean notion of pragmatics, where adhering to the *Cooperative Principle* and corresponding maxims prevents unintended conversational implicatures on the listener side. Here, *adequacy* and *efficiency* largely correspond to the Maxim of *Quantity* (Grice, 1975).

**Figure 1:**
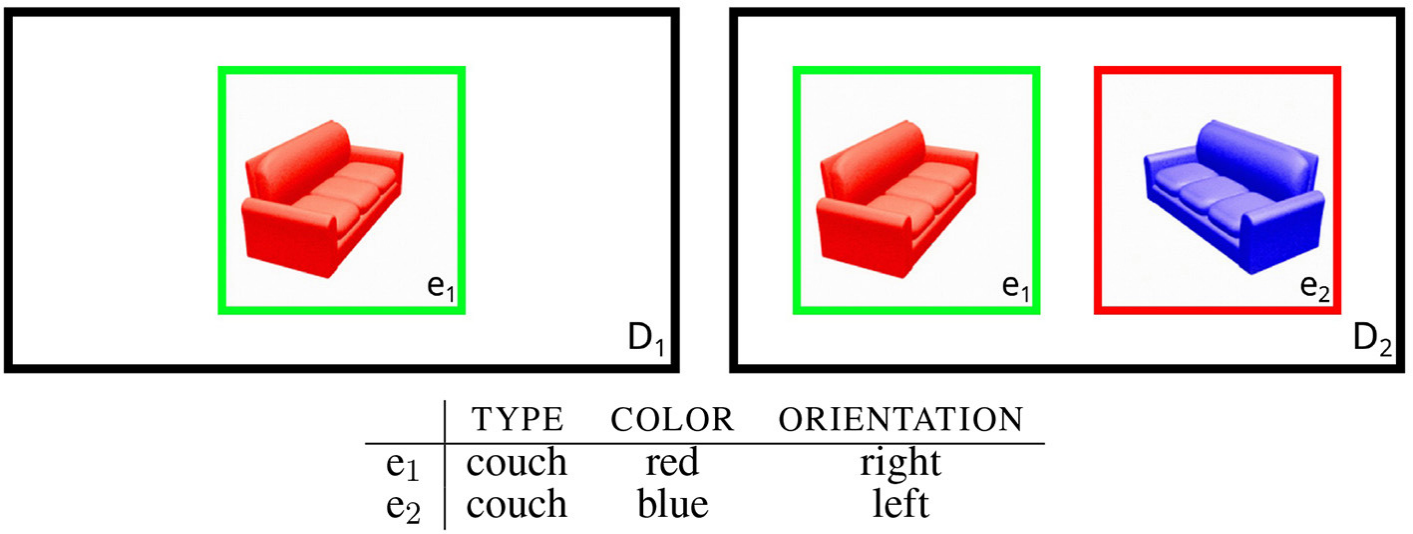
Example for REG settings with images from the TUNA corpus (van Deemter et al., 2006). *e*_1_ is the intended referent in both cases. In domain *D*_1_, it is sufficient to select e.g., the TYPE property. In domain *D*_2_ further properties have to be selected in order to rule out the distractor *e*_2_.

**Figure 2:**
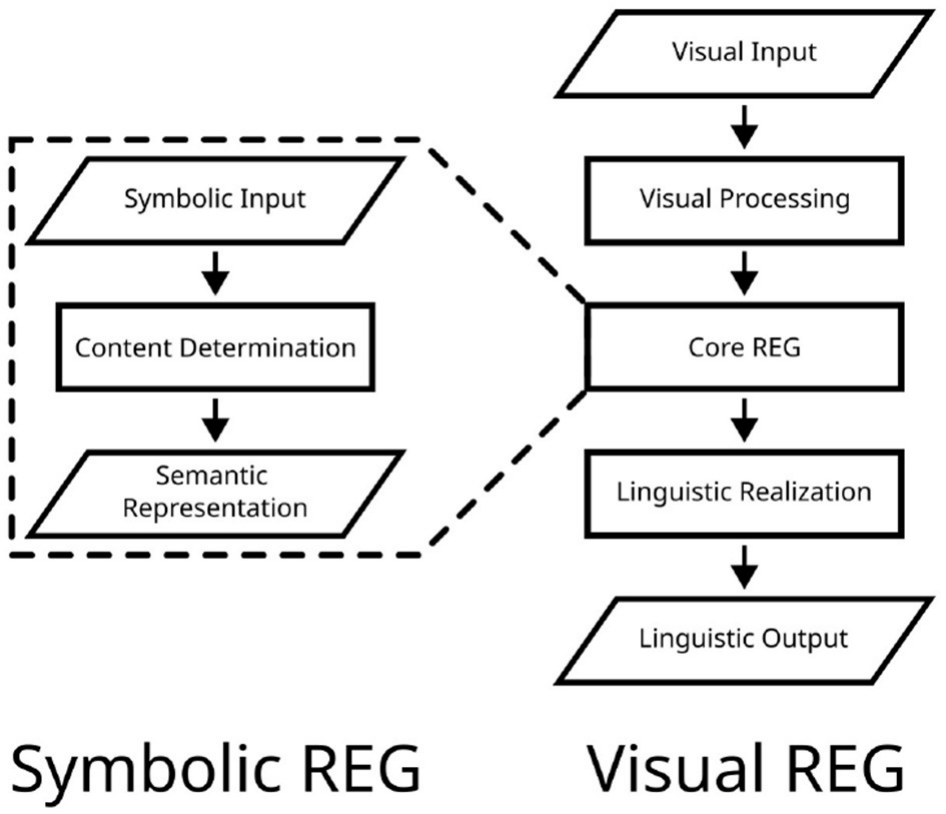
Illustration of general processing stages considered in symbolic **(left)** and visual REG **(right)**. Content determination (selecting a set of distinguishing features from symbolic input representations) is at the heart of the core formulation of symbolic REG. Visual REG comprises all components of symbolic REG, supplemented by visual processing and linguistic realization.

The full process of generating distinguishing descriptions is thought of as involving at least two conceptual processing stages, i.e., *content determination* (deciding on the semantic properties to be expressed) and *linguistic realization* (formulating the selected properties into natural language). However, much work on REG focused on the semantic and pragmatic aspects of the task, i.e., content determination, while addressing realization more cursorily or arguing for the use of general surface realizers (Krahmer et al., 2003; Krahmer and van Deemter, 2019). During content determination, a set of semantic features has to be selected, which collectively apply to the referential target, but not to any of the *distractor* objects in the same domain, therefore ruling out potential competitors during reference resolution. Commonly, this relies on knowledge bases which contain symbolic representations for objects in a given domain (cf. Figure 1 as an example). Thus, generally, the core formulation of the REG task can be considered as including three main components (illustrated in Figure 2, left): (a) an input representation containing symbolic information for objects in the domain, (b) the content determination stage, and (c) an output representation containing the semantic features selected for linguistic realization.

Formally, for a given domain *D* = {*e*_1_, , *e*_2_, ..., *e*_*n*_}, where *e*_1_, , *e*_2_, ..., *e*_*n*_ are entities in *D*, every object *e*_*i*_∈*D* is defined in terms of a set of *properties* *P*_*e*_*i*__. Every *p*∈*P*_*e*_*i*__ has the form 〈*Attribute, Value*〉, where e.g., 〈COLOR, *red*〉∈*P*_*e*_*i*__ indicates that *e*_*i*_ has the attribute *color* with the value *red*. The *context set* *A* is the set of objects the hearer is believed to be attending to, here we assume that *A* = *D*. The content determination stage returns abstract representations of the semantic contents of the expression to be generated, i.e., sets of properties deemed to adequately and efficiently identify the respective referents. Hence, for the referent *r*∈*A* , a successful referring expression *S*_*r*_ is defined as a set of properties which collectively applies to *r* but not to any of the distractors in the *contrast set* *C* = *A*−{*r*} (cf. Dale and Reiter 1995). As an example, Figure 1 shows two domains (*D*_1_ and *D*_2_), which build on a shared knowledge base. In both domains *r* = *e*_1_ is the intended referent (marked green in the illustration). While *e*_1_ is the only object occurring in *D*_1_, *D*_2_ includes *e*_2_ as an additional distractor (marked red). Therefore, *S*_*r*_ = {〈TYPE, *couch*〉} (“the couch”) identifies *r* in *D*_1_, whereas in *D*_2_ other properties are needed to rule out *e*_2_. Possibilities include *S*_*r*_ = {〈TYPE, *couch*〉, 〈COLOR, *red*〉} (“the red couch”) or *S*_*r*_ = {〈TYPE, *couch*〉, 〈ORIENTATION, *right*〉} (“the couch facing right”), assuming that TYPE is always selected.

The Incremental Algorithm (*IA*, Dale and Reiter 1995) is generally regarded as the most influential approach to content determination and has been the basis for an extensive body of subsequent work (cf. the following section). The IA iterates through a pre-defined list of attribute types, ordered by preference. If a property rules out any distractors not previously excluded, the corresponding value is added to the output set of properties to be realized. Crucially, the IA doesn't allow for backtracking, retaining all selected properties even if they turn out to be redundant in later iterations. As a result of this, the algorithm is computationally efficient, but doesn't necessarily yield the smallest possible number of attributes—however, importantly, in similar ways as humans, which were found to overspecify certain kinds of attributes, such as color (Pechmann, 1989).

#### 2.1.2. Extensions of symbolic REG

In its core formulation, REG can largely be considered a self-contained task, in which reference is isolated from confounding factors ubiquitous in natural communication. However, this raises the question of whether the resulting algorithms are *complete*, i.e., whether they are capable of producing an adequate description in a given situation, whenever such a description exists (van Deemter, 2002). Generally, the core REG formulation has been limited to relatively simple cases of reference, including restrictions to (a) one-place predicates, (b) single referents instead of sets of targets and (c) domains with limited extend (van Deemter, 2016; Krahmer and van Deemter, 2019). Some of those restrictions have been lifted in subsequent work.

One of the extensions of foundational REG approaches concerns the number of entities to be referred to. Whereas the core formulation of the task covers reference to single objects, **referring expressions for sets of multiple targets** are ubiquitous in human communication. This has been investigated in several regards: While some works focus on formal logical aspects (Gardent, 2002; van Deemter, 2002; Horacek, 2004), another line of research points to more general ways in which REG algorithms have to be adapted for generating references to multiple targets (Gatt and van Deemter, 2006, 2007). Here, the authors argue for *conceptual coherence* as a further requirement for referring to sets of targets, stating that co-referents should be categorized in similar ways, in order to avoid unintended inferences by the listener. For example, whereas “the student and the Italian” reflects incoherent perspectives, “the Italian and the Maltese” satisfies the constraint, leading to a more felicitous description. To this end, the authors propose additional representations of coherence, i.e., the degree of semantic relatedness between pairs of nouns, based on co-occurrence in large text corpora.

In **relational descriptions** like “the book on the table,” the referent (*book*) is identified via its relationship to other objects in the same domain (*table*). The objects in relation to which the target is described are called *relata* (Krahmer and Theune, 2002) or, focusing on spatial relations, *landmarks* (Kelleher and Kruijff, 2006). Initial approaches for generating relational descriptions have already been proposed in early stages of the classical REG task (Dale and Haddock, 1991a,b) and refined in later works (Krahmer and Theune, 2002; Krahmer et al., 2003; Kelleher and Kruijff, 2006; Areces et al., 2008). Generating relational expressions requires the REG systems to be adapted in several ways: Generally, the property sets defining objects in a domain need to be supplemented by n-ary relations, such as the two-place predicate *on*(*e*_1_, *e*_2_) (“on,” where *e*_2_ is the relatum to *e*_1_), in addition to one-place predicates like *red*(*e*_1_) (“red”). Conceptually, this can be seen as extending the object property sets with predicates of higher arity, interconnecting co-occurring objects at the level of representation (although the extension is sometimes implicit). Complementing this, content determination itself has to be adapted for relations, e.g., by allowing for recursion: In order to successfully refer to an object via another, the relatum has to be identified first.

Finally, as knowledge bases were manually compiled in foundational work on REG, domain representations comprised relatively small numbers of co-occurring objects. However, in more realistic scenarios such as REG in discourse (Krahmer and Theune, 2002; Belz et al., 2010) and visual environments (Kelleher and Kruijff, 2006), domains can be significantly larger. This poses the threat of *combinatorial explosion*, and therefore requires ways to restrict the context set to objects which are contextually relevant. As a possible solution, **prominence** or **salience** values (based on grammatical features, discourse history or visual properties) can be assigned to domain entities, in order to determine sets of relevant distractors for content determination. This can be seen as a reinterpretation of communicative success: Instead of ruling out all objects in the domain apart from the target, the generated expression has to exclude only distractors with similar or higher salience, leaving the referent as the most salient entity as described by the expression (Krahmer and Theune, 2002). The same principle can be applied to relata or landmarks, where salience allows to restrict the set of candidate objects for relational descriptions, facilitating both the computational load during generation as well as the accessibility of the resulting description (Kelleher and Kruijff, 2006).

### 2.2. Visual REG

Foundational work on symbolic REG has paved the way for computational models of linguistic reference by formulating reference as a largely self-contained problem. However, whereas certain linguistic restrictions have been targeted by subsequent work, other limitations remain. This crucially includes the *modality* of input representations: Visible objects are commonly used as a prime example for targets of referring expressions (e.g., Dale and Reiter 1995) and later works specifically revolve around REG in visual (Kelleher and Kruijff, 2006; Mitchell et al., 2013) or three-dimensional environments (Kranstedt and Wachsmuth, 2005). However, the reliance on symbolic information largely prohibits the direct application of REG systems to natural visual inputs (but see Chamorro-Martínez et al. 2021 for a hybrid approach where symbolic properties are extracted from natural images) and leads to the *Symbol Grounding Problem* as described by Harnad (1990): The meanings of symbol tokens in purely symbolic systems are *parasitic*, as they merely rely on the meanings of other symbols without being grounded in e.g., perceptual information. Crucially, the process of visual grounding is associated with a variety of problems and uncertainties, both regarding perception itself and the association of perceptual impressions and symbolic tokens (as e.g., reflected in research on object detection and classification, cf. Zaidi et al. 2022 for a recent survey). For linguistic interaction in shared visual environments, additional problems arise, such as perceptual mismatches between interlocutors (cf. Fang et al. 2013, 2014, 2015 for related work in situated dialog with artificial agents).

**Figure 3:**
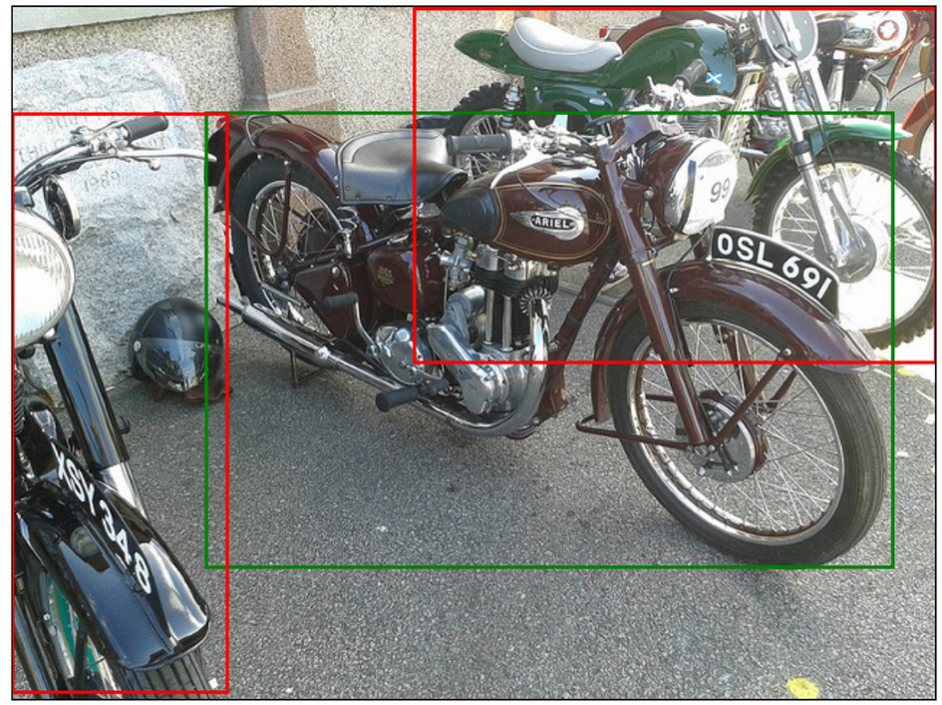
Example from RefCOCO, referential target is marked green. Annotations: “largest bike in the middle”; “bike with 691”; “burgundy bike in center.” Image: 1953 Ariel NH by Graham Robertson, licensed under CC BY 2.0.

In recent years, the availability of large-scale vision and language corpora such as RefCOCO (Kazemzadeh et al., 2014) and more general advances in Computer Vision and neural language modeling have alleviated some of these problems, allowing to extend the REG task to more natural inputs like Figure 3. In this *visual REG* paradigm, the goal is to generate descriptions using raw visual representations of objects in natural images (Mao et al., 2016; Yu et al., 2016, 2017; Zarrieß and Schlangen, 2016, 2018, 2019; Liu et al., 2017, 2020; Luo and Shakhnarovich, 2017; Li and Jiang, 2018; Tanaka et al., 2019; Kim et al., 2020; Panagiaris et al., 2020, 2021; Schüz and Zarrieß, 2021; Sun et al., 2022).

Generally, this task can be considered an *image-to-text* generation problem. Following Mao et al. (2016), it can be defined as determining


$$\begin{array}{l} \underset{S\in V^{*}}{\arg\max}\left(p\left(S|R,I\right)\right) \end{array}$$

where *S* is a sentence (i.e., a string over the vocabulary *V*), *I* is an image, and *R* is a region in *I* containing the referential target. *p*(*S*|*R, I*) is a distribution over candidates for *S*, which are scored in terms of their ability to (a) truthfully describe *R* and (b) identify the target against any other objects depicted in *I*.

From a technical perspective, this line of work is closely related to Image Captioning (Vinyals et al., 2015), although differing in the size of the visual inputs (image regions vs. global images). Neural REG models are generally trained end-to-end and follow the Encoder-Decoder scheme, where the raw visual input is first transformed into intermediate representations by an image encoder and then passed to a decoder, which autoregressively generates linguistic descriptions for depicted entities.

In general, visual REG has largely adopted the task framing of symbolic approaches, i.e., REG remains a self-contained task primarily concerned with enabling identification. Accordingly, much of the research literature revolves around methods to optimize the discriminative power of generated expressions through modifications at different modeling stages. Existing approaches can be roughly divided into two classes: While some work aims at *increasing informativeness* by simulating listener behavior, others focus on *distilling relevant information* from visual inputs or refining the representations themselves. Some approaches are combinations of both categories.

For the first category, different ways have been suggested to increase pragmatic informativity by adapting to simulations of how the generated utterances would be understood by a listener. To this end, as a supplement to the commonly used likelihood-based training objectives, Mao et al. (2016) introduce the *Maximum Mutual Information (MMI)* objective, which penalizes the model if it generates expressions that also apply to other objects in the same image. Schüz and Zarrieß (2021) follow the same intuition, although focusing on the inference stage: Here, they test a set of decoding methods which include probabilistic models of listener behavior, in order to select lexical entries that apply to the target but not to distractors. Other works incorporate training signals from comprehension modules (Luo and Shakhnarovich, 2017) or reinforcement agents (Yu et al., 2017), which convey information about whether the generated description allows to identify the intended referential target.

In contrast to this, other works attempt to increase the informativeness of generated expressions by adapting the visual representations of targets and their surroundings taken as input by the generation models. Stressing the importance of contextual information, Yu et al. (2016) augment the input representations with *visual comparisons*, i.e., information about differences between similar objects in terms of their visual appearance as well as relative positions and sizes. Relating to the importance of attributes in symbolic REG, Liu et al. (2017) use the output of a separate classifier, which predicts symbolic attributes for depicted objects, as additional features for their generation model. Liu et al. (2020) use the same attribute information to guide their model's visual and textual attention, in order to emphasize pragmatically informative contents. Aiming at more comprehensive context representations, Li and Jiang (2018) propose a method for progressively encoding contextual objects using a *visual context LSTM* to allow the model to select the relevant contextual information. Tanaka et al. (2019) use target-centered weighting in combination with attention devices to improve their context representations. In addition, they utilize a reinforcer component to optimize their model's ability to generate unambiguous expressions which are also easy to understand, using annotations of the required times to resolve references in a self-created dataset. Striving for more fine-grained visual representations, Kim et al. (2020) compute the visual differences between targets and more immediate neighbor objects and use attention devices to restrict their context representations to relevant distractors. Finally, Sun et al. (2022) build on grid segmentations of the input images and use Transformer cross-attention to learn joint representations of the target object and the context, without relying on annotated or predicted object segmentations.

### 2.3. Differences between symbolic and visual REG

As described in the previous section, the symbolic and visual REG paradigms differ substantially in the respective input modalities and dominant modeling paradigms. However, there are further differences. For example, with regard to required stages of processing, symbolic REG mainly revolves around content determination. In visual REG, however, it is necessary to first segment and interpret the low-level inputs, ultimately extracting the relevant information about depicted objects and their surroundings. In addition to this, whereas linguistic realization was mostly left implicit in symbolic REG, it has to be carried out in visual REG to produce natural language descriptions. Therefore, on a conceptual level, the visual REG task can be seen as encompassing the processing stages of the symbolic REG task, complemented by both prior (i.e., visual processing) and subsequent stages (i.e., linguistic realization, cf. Figure 2). In practice, however, the boundaries between processing stages are fuzzy, due to the connectionist nature of neural REG models.

The addition of further processing stages brings pragmatic constraints into play, which go beyond the Gricean Maxim of Quantity. First, a particular challenge with raw visual inputs is the *recognition* of depicted elements: In symbolic REG, perfect knowledge about entities in the domain is commonly assumed, essentially guaranteeing the truthfulness of generated descriptions (setting aside e.g., cases of vague properties, Horacek 2005). In visual REG, however, false classifications can result in erroneous descriptions, if a visible referent is identified as the wrong kind of object (e.g., a *table* instead of a *chair*, Zarrieß and Schlangen 2016, 2019). From a pragmatic perspective, this can be seen as violations of the Gricean Maxim of *Quality* (Grice, 1975; see also Dale and Reiter, 1995). In addition to this, given the high complexity of natural images, Tanaka et al. (2019) and Kim et al. (2020) argue for *comprehensibility* as an additional criterion for visual REG, in line with the Gricean Maxim of *Manner* (“Be perspicuous,” Grice 1975).

Finally, further changes caused by perceptual inputs and natural language outputs concern the judgement of referential success in visual REG. Whereas symbolic representations allow to clearly determine whether a generated expression unambiguously identifies the intended target, it is virtually impossible to confidently extract all the information contained in natural visual inputs, making it a great challenge to identify which subset of properties are uniquely true of a referent. As a consequence, referential success is evaluated in terms of whether generated expressions are unambiguous *enough* to identify a referent, with referential success assessed either through human validation (e.g., Yu et al. 2016, 2017) or models performing the inverse task of referring expression comprehension using the generated descriptions (Schüz and Zarrieß, 2021). Complementing this, evaluation metrics from e.g., image captioning are commonly used, comparing the generated expressions to ground-truth descriptions produced by humans.

Therefore, although sharing the general task framing, visual REG exhibits a range of differences and additional challenges in comparison to symbolic REG. This includes the widespread adaptation of neural modeling, as well as the scope of the task, where raw visual inputs require stages of visual processing and natural language outputs prevent the maintenance of sharp boundaries between content determination and linguistic realization. Relatedly, with perceptual inputs, the truthfulness of generated descriptions becomes a considerable challenge, and visual salience affects the comprehensibility of generated descriptions. Finally, the higher complexity of input and output representations demands for changes in evaluation, where human or automatic resolution performance as well as likelihood-based metrics have largely replaced the crisp success criteria in symbolic REG.

## 3. Types of context in symbolic REG

In Sections 2.1.1, 2.1.2, we characterized the core formulation of symbolic REG and highlighted a number of directions in which the task has been extended. In the following, we want to take a different perspective: A core assumption in REG is that generating identifying descriptions does not only depend on properties of the referential target, but also on the *situational context*, i.e., other objects co-occurring in a shared domain with the referent. Here, we will analyze how contextual information is integrated and used in the core formulation for symbolic REG and different extensions to this paradigm. Through this, as a framework for the remaining article, we derive a taxonomy of *categories of contextual integration* (or *types of context*), which differ in the ways in which contextual information affects content determination (described in terms of *semantic forces* exerted on the selection process, cf. Gatt and van Deemter 2007) and the pragmatic objectives which are supported by the integration.

### 3.1. Distractor context

**Figure 4:**
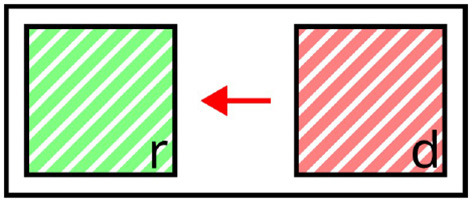
Illustration of distractor context. The distractor *d* provides negative context (red arrow) to the referent *r*. Valid expressions: “the *green* square,” “the *left* square” (affected attributes highlighted).

In the core formulation of REG (cf. Section 2.1.1), co-occurring *distractors* represent the primary form of situational context. For example, in Figure 4, the red square (*d*) acts as a distractor when referring to the green square (*r*). Here, during content determination, selected properties are mainly assessed in terms of whether they represent the right amount of information, as formulized in the constraints of *adequacy* (whether a referring expression contains enough information to identify the target) and *efficiency* (whether it is not overly informative, Dale and Haddock 1991a). Crucially, both constraints depend on co-occurring objects: A description which satisfies both principles in one context can be ambiguous or triggering unintended implicatures in others, in line with the Gricean Maxim of *Quantity*.

For *adequacy* to be satisfied, the description has to express properties which are included in the target property set, but rule out all distractors in the contrast set. To this end, in principle, pairwise comparisons have to be made between the property sets of the target and all distractors in the contrast set, in order to select at least one property for each distractor which rules out the respective object. Formally, the adequacy requirement can be decomposed into two sub-constraints, requiring a generated expression to (1) apply to the referent, but (2) not to any of the distractors in the contrast set (Dale and Reiter, 1995). Hence, for a given referent *r* with properties *P*_*r*_ and a contrast set *C* = {*d*_1_, *d*_2_, ..., *d*_*n*_} with properties *P*_*d*_ for every distractor *d*∈*C*, an expression *S* is adequate iff


$$\begin{array}{l} \forall p\left[p\in S\;\Rightarrow \;p\in P_{r}\right] \end{array}$$

and


$$\begin{array}{l} \neg \exists d\left[d\in C\wedge \forall p\left[p\in S\;\Rightarrow \;p\in P_{d}\right]\right] \end{array}$$

For the latter to be true, the following condition has to be met for every distractor:


$$\begin{array}{l} \forall d\left[d\in C\;\Rightarrow \;\exists p\left[p\in S\wedge p\notin P_{d}\right]\right], \end{array}$$

where *S*⊆*P*_*r*_.

As a result, the influence of an individual distractor object *d* to the process of content determination can be seen in (a) requiring the selection of at least one property from the difference set *P*_*r*_ \*P*_*d*_, contrasting the target from this particular distractor, and (b) providing the speaker with the necessary information to select {*p*|*p*∈*S*∧*p*∉*P*_*d*_}, thus making *S* a distinguishing description of *r* with respect to *d*.

The impact of distractors on the selection of target properties can be described in terms of certain *semantic forces*: First, as a result of the adequacy constraint, distractors enforce the selection of properties which apply to the target, but not to themselves. In addition, the efficiency constraint penalizes the selection of properties shared by the target and any distractor, unless they rule out any other object in the contrast set. Taken together, distractors steer the generation process toward properties that are not contained in their own sets of defining properties, as all distractor properties are inherently disfavored in the selection for the final expression, due to *adequacy* and *efficiency*. Therefore, in terms of the semantic forces they exert on the generation process, distractors can be seen as *negative context*. This is illustrated in Figure 4, where the distractor *d* requires discriminative properties like *green* or *left* to be included in expressions referring to *d*, but adding the common property *striped* would violate the efficiency constraint.

### 3.2. Perspective cues

**Figure 5:**
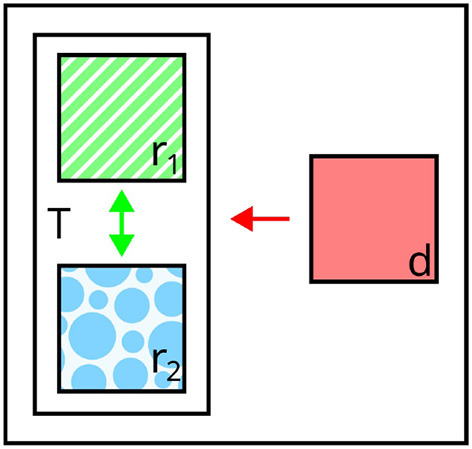
Illustration of perspective cues. When referring to *T* = {*r*_1_, *r*_2_}, the co-referents require coherent perspectives, providing reciprocal positive context (green arrow). Valid expressions: “the *green* and the *blue* square,” “the *striped* and the *dotted* square” (affected attributes highlighted).

While negative context in the form of distractors is crucial for achieving discriminability, other types of context can be associated with semantic forces that are diametrically opposed. Crucially, this involves further pragmatic constraints: For example, Gatt and van Deemter (2006, 2007) propose the *Conceptual Coherence Constraint* for generating references to multiple targets (cf. Section 2.1.2). This criterion (“As far as possible, conceptualize elements of a plurality in similar ways,” Gatt and van Deemter 2007) requires mechanisms for integrating context in symbolic REG, which maintain consistent perspectives across co-referents, emphasizing conceptual *similarities* instead of *differences* between co-occurring objects in the generation process. For instance, when referring to *r*_1_ and *r*_2_ in Figure 5, “the *green* and the *blue* square” would be a coherent description, whereas “the *striped* and the *blue* square” would be equally discriminative, but reflect different conceptual perspectives.

Gatt and van Deemter (2007) focus on plural descriptions of the logical form λ*x*(*p*(*x*)∨*q*(*x*)), realized as descriptions of the form “the *N*_1_ and the *N*_2_.” Given this, the extension of *S*_*T*_ uniquely identifying a set of referents *T* = {*r*_1_, *r*_2_, ..., *r*_*n*_} is the union of the individual referents ⟦*S*_*T*_⟧ = *r*_1_∪*r*_2_∪...∪*r*_*n*_. Hence, the semantic content of *S*_*T*_ can be decomposed into sets of properties identifying each of the individual referents, i.e., *S*_*T*_ = *S*_*r*_1__∪*S*_*r*_2__∪...∪*S*_*r*_*n*__, where ⟦*S*_*r*_*i*__⟧ = *r*_*i*_∈*T*.

The Conceptual Coherence Constraint is satisfied in the case of *Maximal Coherence*: “A description *S* is maximally coherent iff there is no description *S*′ coextensive with *S* such that *w*(*S*)>*w*(*S*′)” (Gatt and van Deemter 2007, variable names adapted). Here, *w*(*S*) indicates the *total weight* of *S*, calculated in terms of the number and relatedness of perspective clusters reflected in the description. Given the semantic structure of *S* and the global scope of *w*, satisfying Maximal Coherence amounts to determining the set of properties *S*_*T*_ which has the lowest total weight, i.e.,


$$\begin{array}{l} \underset{S_{T}}{\arg\min}\left(w\left(S_{T}\right)\right) \end{array}$$

where


$$\begin{array}{l} w\left(S_{T}\right)=w\left(S_{r_{1}}\cup S_{r_{2}}\cup ...\cup S_{r_{n}}\right), \end{array}$$

while maintaining *adequacy*, i.e., *S*_*T*_ uniquely describes *T*.

This indicates two important differences compared to the ways in which distractor context is integrated in the generation process: First, while the integration of distractor context is characterized by pairwise comparisons between the target and individual objects in the contrast set (i.e., individual distractors are independent of each other), conceptual coherence intertwines the choice of properties for all entities in the referent set. This is due to the scope of *w*(*D*): The total weight of generated perspectives is determined globally over *S*_*T*_, leading to every *r*∈*T* (reciprocally) affecting the choice of equally discriminative properties for all *r*′≠*r*∈*T*. Second, here, the semantic forces are opposed to the ones exerted by distractors. Following Gatt and van Deemter (2007), choosing different combinations of properties reflects different categorizations of an entity, implicitly involving the adaptation of a certain conceptual perspective—in this light, members of referent sets can be described as *(conceptual) perspective cues* for their co-members, as the ways in which they are conceptualized affect the adequacy of conceptual perspectives for all other members of the referent set. Importantly, whereas distractors guide content determination toward properties expressing *differences* between them and the targets, the Conceptual Coherence Constraint enforces *similarity* between the properties selected for the individual co-referents, i.e., favoring combinations of properties associated with the same or related perspectives, thus minimizing the total weight. Therefore, in contrast to the *negative* context as represented by distractor context, perspective cues provide *positive* context to the process of REG.

However, while the semantic forces are opposed, they do not apply at the same levels: Regarding distractor context, the expression has to contain at least one property, for which target and distractor have different *values* (or, arguably, which is not specified for the distractor at all). In contrast to this, conceptual coherence rather affects the choice of *types of attributes*, i.e., Maximum Coherence demands the selection of related attributes for co-referents, irregardless of their respective values. For example, in Figure 5, coherent referring expressions for *T* = {*r*_1_, *r*_2_} include “the *green* and the *blue* square” and “the *striped* and the *dotted* square,” reflecting the common perspectives *color* and *pattern*, respectively. In this sense, positive and negative semantic forces complement each other—in fact, sets of distractors and perspective cues can intersect, for example in “the Italian and the Maltese,” where the co-referents are referred to using the same attribute type, but different values (thus allowing for individual identification). Similarly, whereas accounting for distractors is necessary to satisfy the Gricean Maxim of Quantity, this is arguably not the case for perspective cues. However, utterances with incoherent perspectives are perceived as *marked* by the listener, triggering reasoning processes about the ways in which the speaker categorizes an object. In this sense, considering perspective cues might primarily correspond to the Maxim of *Relevance* (Grice, 1975), i.e., violations of the Conceptual Coherence Constraint can cause the listener to infer that particular aspects of a referent are relevant to the message, even if unintended by the speaker.

### 3.3. Relata and landmarks

A further type of contextual integration are *relata* or *landmarks*, in relation to which referents are described in relational expressions such as “the book on the table” (where *table* is the relatum to *book*, cf. Section 2.1.2). Generally, generating relational expressions requires the extension of object representations by properties expressing n-ary relations between multiple entities, in addition to the one-place predicates as used in the core formulation. Relations can be integrated a priori into the property sets (Dale and Haddock, 1991a,b; Krahmer and Theune, 2002; Krahmer et al., 2003) or added successively during iteration through a hierarchy of possible types of relations (Kelleher and Kruijff, 2006).

Formally, a property set *P*_*e*_ for an object *e* in domain *D*, which includes unary properties as well as n-ary relations, can be decomposed into the union of multiple subsets containing predicates of specific arity, i.e., *P*_*e*_ = *P*_1_*e*__∪*P*_2_*e*__∪...∪*P*_*n*_*e*__, where *P*_1_*e*__ contains 1-place predicates, *P*_2_*e*__ contains 2-place predicates, and so on. For 1-place predicates, contextual information about co-occurring objects is not included in the property set, as information about *e* alone suffices for determining whether a given property applies to it:


$$\begin{array}{l} P_{1_{e}}=\left\{\langle A,V\rangle |e\;has\;the\;value\;V\;for\;attribute\;A\right\} \end{array}$$

This is different for predicates with higher arity, i.e., relations between objects. Here, information about co-occurring entities has to be integrated to determine whether a given relation exists between them. For the case of 2-place predicates:


$$\begin{array}{l} P_{2_{e}}=\left\{\langle A,R\left(e,e^{\prime }\right)\rangle |e^{\prime }\in D\wedge \langle e,e^{\prime }\rangle stand\;in\;relation\;R\right\} \end{array}$$

(cf. Krahmer and Theune 2002).

In this respect, information about an object *e*′ as context for *e* is integrated (a) to determine existing relations where *e*′ is a relatum to *e* and (b) at the level of representation, where *e*′ is an argument for those relations, which can be selected as properties during content determination.

**Figure 6:**
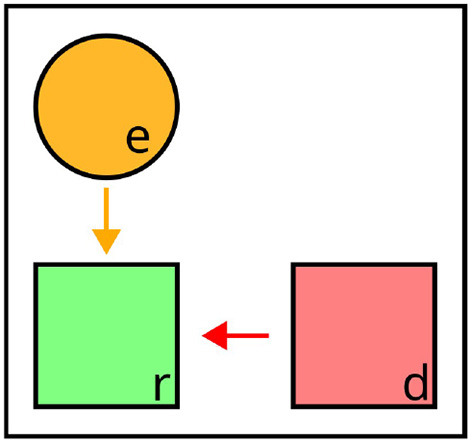
Illustration of context in relational descriptions. *e* acts as a relatum to the referent *r* (yellow arrow), supplementing the property set with n-ary relations. Valid expression: “the square *under the circle*” (affected attributes highlighted).

The integration at the level of representation points to general differences in comparison to both distractors and perspective cues. Whereas these types of context could be integrated in a distinction between *positive* and *negative* semantic forces, relata or landmarks do not (directly) affect the selection of target properties during content determination, but rather constitute pieces of information included in the property sets of co-occurring objects, which can themselves be used for reference. In this sense, they can be seen as *instrumental* to positive or negative semantic forces: As both operate on object properties, and relata or landmarks are used as arguments for relations extending the property sets of other objects in the domain, they increase the space of possible dimensions along which both positive and negative forces can be exerted. For example, in Figure 6, *e* is a relatum for *r* as the referential target, allowing for *under*(*r, e*) to be used to rule out distractor *d* (“the square under the circle”).

As a result of this, relata and landmarks also differ from distractors and perspective cues with respect to the pragmatic objectives they facilitate. The types of context discussed above were argued to contribute to satisfy the Gricean Maxims of Quantity and Relation, respectively. Given our assumption that relata and landmarks are instrumental to other semantic forces, they naturally are involved in this. However, Viethen and Dale (2008) argue that there are cases in which relational expressions are preferred over expressions consisting of one-place predicates, e.g., when relata are highly prominent, making corresponding relations salient properties of the target. In such cases, incorporating this type of context could additionally be seen as a means to satisfy the Gricean Maxim of *Manner*, in accordance with Tanaka et al. (2019) and Kim et al. (2020)'s work on visual REG (cf. Section 2.2).

### 3.4. Prominence and salience

Finally, as discussed in Section 2.1.2, notions of *prominence* and *salience* have been proposed to determine whether co-occurring objects are relevant distractors (Krahmer and Theune, 2002) or landmarks (Kelleher and Kruijff, 2006). For the former, the notion of *adequacy* is adapted—other entities only have to be ruled out, if they are at least as salient as the referential target. Implicitly, this amounts to the adoption of dynamic context sets, which depend on the target and are restricted to elements with greater salience than the intended referent:


$$\begin{array}{l} A_{r}=\left\{e\in D|sw\left(e\right)\geq sw\left(r\right)\right\}, \end{array}$$

where *D* is a domain and *A*_*r*_⊆*D* is a dynamic context set with respect to a referent *r*∈*D*. Membership to *A*_*r*_ is determined by means of a *salience weight* function *sw* which maps entities in *D* to natural numbers, indicating their salience (Krahmer and Theune, 2002).

**Figure 7:**
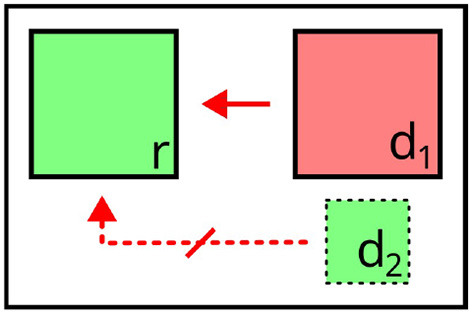
Illustration of prominence/salience. *d*_2_ is not considered as a relevant distractor for *r*, due to its lower salience. Valid expression: “the *green* square” (affected attribute highlighted).

Similar to relata and landmarks, here, the effects on content determination are less overt than for distractors and perspective cues. It even seems debatable whether salience can be considered as a form of *context*, if the relative salience of objects primarily determines whether *they themselves* are considered in the generation process (i.e., the salience of an object does not directly affect other objects). However, by directing attention to themselves, salient objects can also increase the salience of neighboring objects (*implied spatial salience*, Piwek 2009). While salience does not directly affect content determination for co-occurring objects, it is a determinant for the composition of the sets of relevant distractors and possible landmarks. Salient objects can exert negative semantic forces and can be considered as landmarks, whereas objects with low salience are excluded from the generation process. For instance, in Figure 7, *d*_1_ is a relevant distractor when referring to *r*, whereas *d*_2_ is disregarded due to its low salience.

In this light, salience can be seen as *modulating* semantic forces exerted by context objects, without altering their directionality. Here, the modulation is binary (i.e., context entities are excluded entirely if falling beyond a salience threshold), but in probabilistic approaches and natural communication modulations can be expected to be more fluid, i.e., low salience context can still exert semantic forces, although with smaller effect.

### 3.5. Dimensions of context in symbolic REG

In this Section we took another perspective on the core REG formulation and extensions to it, characterizing the different approaches in terms of how they integrate contextual information about co-occurring objects. Through this, we derived a number of types of context:

**Distractor context** is a crucial component of the core formulation of the task. As a result of the adequacy and efficiency constraints, distractor objects exert *negative* semantic forces during the generation process, steering content determination toward properties that do not apply to themselves. In this regard, distractor context is integrated in order to satisfy the Maxim of *Quantity*, i.e., generate expressions which uniquely describe the target without being overly informative. In contrast to this, **perspective cues** can be understood as a form of *positive* context, guiding the expression generation toward similarities between co-referents in plural expressions. While this is generally opposed to the modes of action which can be seen for distractor context, it can be seen as complementary, as it applies to different stages (types of attributes instead of property values). Regarding the Gricean Cooperative Principle, this mainly affects the Maxim of *Relevance*, as conceptual coherence prevents unintended inferences regarding the significance of specific aspects of the referent. **Relata and landmarks** are context objects, in relation to which target objects can be characterized and described. Whereas they do not fit easily into a distinction between positive and negative effects, they complement the generation process in different ways: By supplementing the property sets of co-occurring objects with n-ary relations, they add further dimensions along which both positive and negative context effects can operate, i.e., they are *instrumental* to other semantic forces. In addition, generating relative descriptions involving visually salient landmarks can facilitate reference resolution, in accordance with the Maxim of *Manner*. Finally, the **prominence or salience** of context objects *modulates* the extend to which they exert semantic forces: Whereas it does not directly affect the generation process, it determines the contextual relevance of an object, i.e., the extent to which it affects the generation process in the aforementioned ways.

Importantly, these dimensions of context are not mutually exclusive, as one and the same object can be integrated in different ways. For example, in “the Italian and the Maltese,” the co-referents reciprocally act as perspective cues (providing positive context), but are also considered as distractors (negative context). Similarly, in “the bowl on the table,” the relation *on(x,y)* involves *table* as a relatum, but at the same time rules it out as a distractor (as the relation is not symmetrical).

In the following section, we investigate whether the dimensions of context identified for symbolic REG are reflected in existing approaches for visual REG.

## 4. Situational context in visual REG

As discussed in Section 2.2, the shift to raw visual inputs in REG led to a wider focus for modeling. Whereas symbolic REG builds on high-level representations of objects and domains, low-level visual information is used as input for visual REG. Naturally, this extends to the representation of situational context: Here, information about distractors, perspective cues and landmarks has to be extracted from the *visual context* surrounding the referential target. In this section we will first review the different ways of representing situational context in visual REG and the differences to symbolic REG in this regard. After this, we will return to the types of contextual integration discussed for symbolic REG in the previous section, and analyze whether they are reflected in the visual REG literature.

To the best of our knowledge, there has been no comparative review of the role of situational context in visual REG. However, a large body of related research highlights the crucial importance of visual context in this field. Generally, visual context has been found to facilitate visual tasks across various fields, such as cognitive psychology (Palmer, 1975; Chun and Jiang, 1998; Albright and Stoner, 2002; Bar, 2004; Torralba et al., 2006; Oliva and Torralba, 2007) and Computer Vision (Strat, 1993; Rabinovich et al., 2007; Divvala et al., 2009; Galleguillos and Belongie, 2010; Liu et al., 2019). More closely related to REG, different ways of integrating visual context have been found to increase system performances in the inverse task of Referring Expression Comprehension (Nagaraja et al., 2016; Zhang et al., 2018; Wang et al., 2019). For human reference production, different parameters of visual scenes have been shown to affect form and content of produced utterances, including e.g., spatial structures (Baltaretu et al., 2016; Koolen, 2019), properties of co-occurring objects (Koolen et al., 2015) and visual salience (Fukumura et al., 2010; Clarke et al., 2013a,b, 2015; Vogels et al., 2013), the latter being connected to more general work on how visual context contributes to attention allocation (Oliva et al., 2003; Torralba et al., 2006).

### 4.1. Representations of context in visual REG

Visual context can influence cognitive processes at different levels of abstraction and granularity. For the former, in natural images, contextual information can both consist of *low-level* features, such as textures and local contrast, and *high-level* information about meaningful units, such as objects or scenes (Võ, 2021). Complementary to this, visual context can be both *local* (i.e., extracted from specific parts of the image, such as the immediate surroundings of a visual target) or *global* (extracted from the overall display, Liu et al. 2019). Regarding those dimensions, in symbolic REG, situational context usually consists of high-level information, i.e., sets of properties for objects co-occurring in a domain. The scope of contextual information is usually global, i.e., objects from the entire domain are considered as context (partly because information about the relative position of different objects is not always included in the symbolic representations).

In visual REG, at the beginning of the generation process, situational context generally consists of low-level representations (i.e., color values of pixels in the image), which are used as input for the model encoder. In more detail, however, there are different approaches to input representations, which are sometimes used in combination. First, this includes *global context vectors*, i.e., visual information from the whole image, commonly obtained from pretrained CNN encoders. In Tanaka et al. (2019), the global context is centered around the target using Gaussian weighting; Kim et al. (2020) use a pretrained Faster-RCNN to compute averaged feature vectors of all detected objects in an image. Second, *visual comparisons* express appearance differences between targets and co-occurring objects, optionally restricted to neighbors of the same type (Yu et al., 2016) or high relevancy, as determined by attention logits (Kim et al., 2020). Few works integrate visual context in substantially different ways: Whereas Li and Jiang (2018) sequentially encode detected objects in a *visual context LSTM*, Schüz and Zarrieß (2021) include CNN features of co-occurring objects during inference, based on annotated bounding boxes. In their Transformer-based approach, Sun et al. (2022) rely on grid segmentations of the input images and use cross-attention to learn contextualized representations of referential targets.

### 4.2. Types of context in visual REG

As discussed in Section 2.2, visual REG conceptually comprises the processing stages of the core REG formulation. In addition to this, as neural REG models are trained and evaluated on the basis of crowd-sourced natural language descriptions, a priori restrictions to certain formats of referring expressions are hardly possible—visual REG models should therefore be capable of generating referring expressions with various linguistic devices, including plural and relational expressions (cf. Section 2.1.2). In general, therefore, a comprehensive approach to visual REG should at least encompass all types of context that have been incorporated into the symbolic REG extensions to generate more variant expressions (cf. Section 3). However, the types of context reflected in the visual REG literature are less diverse than expected: As previously described, most works in visual REG have adopted the general task framing of symbolic REG, i.e., enabling identification is stated as the primary objective of the task. In line with this, the roles of visual context largely align with foundational work on REG: For a given referent, surrounding visible objects are mainly factored in as negative distractor context, i.e., as necessary information for the generation of discriminative utterances.

Some works are comparatively clear about the dimensions of context that are relevant to their approaches. Here, the consideration as distractor context is most common. For example, Mao et al. (2016) state that context is critical for the speaker to “differentiate the target object from a collection of alternatives,” which requires them to “reason about how the object differs from its context.” In Yu et al. (2016), different kinds of context representations are designed to amplify differences in visual appearance and location between the targets and co-occurring objects through visual comparisons. In Zarrieß and Schlangen (2016), a model is considered *context-aware* if it avoids ambiguities between descriptions of targets and distractors. Similarly, Schüz and Zarrieß (2021) take “linguistic adaptation to context” as increasing pragmatic informativity by generating expressions which describe the referent but not other objects. Reflecting a wider notion of context, Li and Jiang (2018) argue for more granular and flexible representations, in order to account for the varied relationships between different objects in an image, which are not only important as distractors but also e.g., as relata to further objects.

In other cases the primacy of distractor context is reflected by modeling decisions on different levels of the architecture. As described in Section 2.2, this includes dedicated training objectives (Mao et al., 2016), representations of visual differences (Yu et al., 2016), training signals conveying the referential success from comprehension modules (Luo and Shakhnarovich, 2017) or reinforcement agents (Yu et al., 2017), or decoding procedures aimed at selecting lexical entries that apply to the target but not to distractors (Schüz and Zarrieß, 2021). Despite the considerable differences, all of these approaches share the common trait of aiming at negative semantic forces between targets and co-occurring objects.

Apart from distractor context, notions of prominence and salience are echoed in different approaches to distill relevant information from the input. In contrast to symbolic REG, there are no dedicated values for the relative salience of referential targets and surrounding objects. Instead, REG models include different types of context-driven selection mechanisms, which are learned during the general training. A prime example for this are the attention mechanisms (Bahdanau et al., 2015; Xu et al., 2015), which allow REG models to selectively focus on e.g., visually salient or pragmatically relevant parts of the input representations (Tanaka et al., 2019; Kim et al., 2020; Li et al., 2020; Liu et al., 2020; Panagiaris et al., 2021; Sun et al., 2022), in line with both Dale and Reiter (1995)'s definition of context sets (cf. Section 2.1.1) and more general findings about the role of attention allocation for reference production (see above). In Li and Jiang (2018) the gating mechanisms of the *visual context LSTM* fulfill similar functions.

Finally, for relata and landmarks, Li and Jiang (2018) argue for comprehensive representations of context, in order to capture the rich relationships between visible objects. In addition to this, Tanaka et al. (2019); Kim et al. (2020) highlight the importance of relational descriptions for generating expressions which are not only discriminative but also easy to understand. Importantly, in contrast to related approaches in symbolic REG, these works do not propose dedicated model components to facilitate the integration as relations or landmarks, but argue for improvements in context representations. In addition, Tanaka et al. (2019) directly try to optimize the comprehensibility of the generated expressions during training by using the accuracy and required time of human annotators to resolve the ground-truth expressions as input to a dedicated loss function.

Thus, although visual REG conceptually comprises all extensions of the core REG task, as well as visual processing and linguistic realization (cf. Section 2.2), not all types of contextual integration discussed in Section 3 are equally reflected in the literature. While different kinds of representation have emerged (e.g., global images or visual comparisons), visual context is mainly regarded as a form of negative distractor context, i.e., leveraged to reduce ambiguities in generated descriptions, in line with foundational work in symbolic REG (Dale and Reiter, 1995). In addition to this, parts of the visual REG literature reflect aspects of prior research on relational descriptions as well as prominence and salience. However, to the best of our knowledge, there are no approaches to visual REG that integrate visual context for positive semantic forces, such as those exerted by perspective cues. Likewise, little attention has been paid to the question of how visual context can be used to address the inherent uncertainty of low-level visual representations. Against this background, in the following section, we want to highlight a number of further aspects regarding the integration of visual context in REG.

## 5. Toward a wider notion of context in visual REG

In the previous section, we showed that the wider task formulation in visual REG is seldom reflected in the integration of different types of context. In the following, we will outline two additional ways in which information from the visual context can be utilized to address some of the further challenges arising from the paradigm shift from symbolic to visual REG. First, we will discuss how *scene context* supports the recognition of objects in low-level inputs. Following this, we will explore the role of positive context in visual REG.

### 5.1. Context facilitates recognition: The case of scene context

A major challenge arising from the shift to perceptual inputs in REG is the recognition of depicted objects and entities (cf. Section 4). As described in Zarrieß and Schlangen (2016), recognizing visual objects is a necessary step in visual REG: Before an object can be described to a listener, the speaker must first categorize it. This step can be linked to determining the TYPE attribute of entities as a sub-step in classical REG algorithms, which has been given a privileged role in seminal works (e.g., Dale and Reiter 1995). As discussed earlier, issues in recognition can cause various downstream problems, e.g., violations of the Maxim of Quality if it concerns the target or relata/landmarks, or issues with ambiguity for erroneously classified distractors.

**Figure 8:**
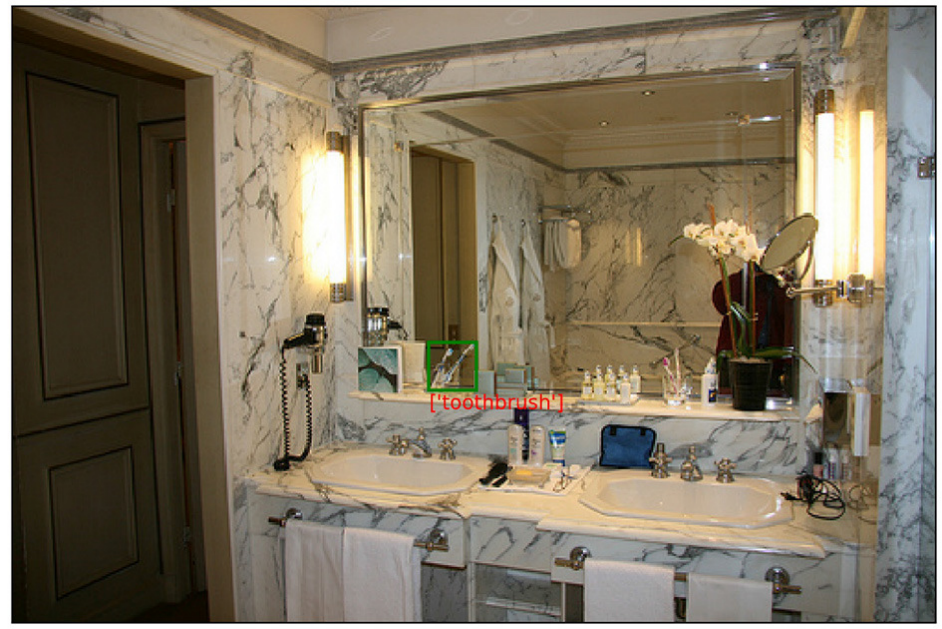
Example from visual genome (Krishna et al., 2017): The *toothbrush* is hard to recognize due to size and occlusion. However, recognition is facilitated by contextual information about surrounding objects and the type of scene. Image: Le Meurice by Langmuir family, licensed under CC BY 2.0.

Object recognition can be especially challenging in cases of deficient visual information, for example if objects are small or partially occluded by other objects (Yao and Fei-Fei, 2010). An example for this can be seen in Figure 8 where the *toothbrush* is hard to recognize due to its size and overlap with other objects. Whereas compensating for this has been an active field of research in related tasks such as referring expression grounding (Wang et al., 2021), it has hardly been investigated for REG yet. Crucially, information from the visual context can be leveraged to alleviate issues with e.g., deficient visual representations, as indicated by a large body of related research from psychology and Computer Vision where contextual information was shown to facilitate the recognition and categorization of visible objects across different tasks (e.g., Oliva and Torralba 2007; Divvala et al. 2009; Galleguillos and Belongie 2010).

Here, the *scene context* (Biederman, 1972; Bar, 2004; Greene, 2013; Pereira and Castelhano, 2014; Võ, 2021) is of particular use. In the real world, objects do not appear randomly, but are characterized by predictable spatial, semantic or functional relations to their surroundings. Learnt knowledge about these regularities in natural scenes can be exploited for visual processing, e.g., to disambiguate the classification of visible objects by priming contextually expected object types. As discussed in Section 4, visual context comes in different flavors and can consist of both global and local features. This also applies to scene information supporting object recognition: Cognitive tasks have been shown to be facilitated by the rough global content of scenes (*gist*, Oliva and Torralba 2006), which can be accessed rapidly and without the need to identify depicted objects. At the same time, more local information about co-occurring objects also supports recognition, as many combinations of objects tend to occur in regular configurations. For this, *anchor* objects (like *stove* or *shower*) are especially significant, as they are highly diagnostic for certain types of scenes (e.g., *kitchen* or *bathroom*) and tend to form hierarchical *phrases* of co-occurring objects (like *pans* or *shampoo bottles*), which commonly have defined spatial relations to their anchors, grounded e.g., in functional relationships (Võ, 2021).

Given this, there are different ways in which scene information can be useful to disambiguate the recognition of objects hard to recognize. First, the *scene type* can be informative as an expectation prior for objects commonly occurring in certain environments. For this, the type of a scene can be determined either on the basis of global features (such as the gist of a scene) or local features (such as objects which typically occur in particular scenes). Second, information about object co-occurrence can be leveraged more directly, either by treating scenes as bags-of-objects or focusing on proximate anchor objects. Some of these contextual cues are exemplified in Figure 8, where recognizing the *toothbrush* is facilitated in several ways: First, the global scene is identifiable as a *bathroom*, which generates an expectation bias for e.g., hygiene products. Second, the target object is in close proximity of other objects it typically co-occurs with, such as the *sink* which serves as an anchor for surrounding objects. In a general visual REG pipeline (cf. Figure 2), the processes that support object recognition by incorporating scene context would mostly be located at the level of visual processing, i.e., prior to linguistic processing itself. Importantly, however, improvements in visual processing could provide more comprehensive and reliable representations of visible entities, ultimately leading to more truthful and pragmatically effective utterances. For example, in Figure 8, improving the recognition of the *toothbrush* would, on the one hand, allow more precise descriptions of the object itself. On the other hand, it could also support the identification of other entities by including the object as a relatum, e.g., “the glass with the blue *toothbrush* in it.”

To the best of our knowledge, scene context has not yet been integrated into visual REG models (but see Cafagna et al. 2021 for the inverse task of grounding descriptions in images). However, it is save to assume that leveraging this kind of context is a challenging modeling problem in itself. Most notably, extracting the relevant information from scenes either requires further stages of visual processing (e.g., in order to classify types of scenes, before this information can be used to compensate for recognition problems) or recursive approaches to recognition, if objects are to be identified through their co-occurrence with other objects. Hence, incorporating scene context involves a wide range of modeling decisions and challenges, the more detailed examination of which we leave for future research. Generally, however, we strongly advocate for a more focused consideration of object recognition as a sub-problem of visual REG and see great potential in exploring different ways of integrating scene context, in order to make reference generation under visual uncertainty more robust.

### 5.2. Perspective cues in visual context

In Section 3.2, we discussed *(conceptual) perspective cues* as a form of positive context where similarities between co-referents are emphasized in the generation process, leading to descriptions that are coherent in terms of the conceptual perspectives for all targets in the referent set. Importantly, conceptual perspectives relate to alternative ways of categorizing objects and entities, as opposed to *visual* perspective in the sense of different viewpoints in three-dimensional scenarios (Herbort et al., 2021). For symbolic REG the choice of conceptual perspectives is investigated in Gatt and van Deemter (2006, 2007). However, the restriction to descriptions of the format “the *N*_1_ and the *N*_2_” complicates direct comparisons with visual REG, as the annotations in e.g., RefCOCO are mostly focused on single objects and rarely correspond to this description format.

**Figure 9:**
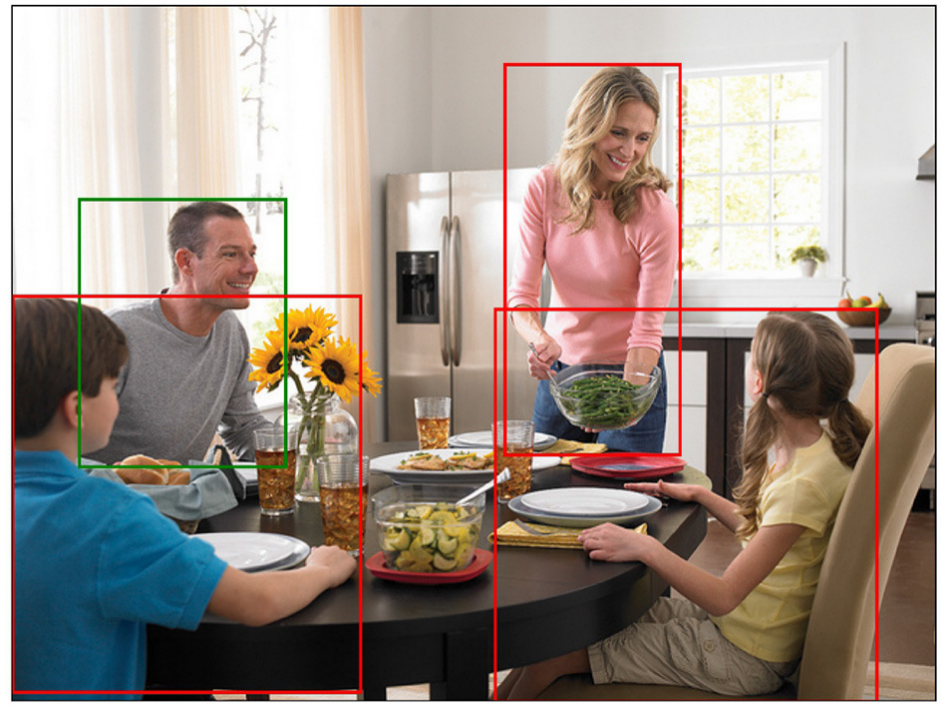
Example from RefCOCO+, referential target is marked green. Annotations: “father”; “dad”; “man.” Image: Glass food storage container - Family Dinner by Rubbermaid Products, licensed under CC BY 2.0.

Still, the general notion of positive context appears to be highly relevant for visual REG. This can be seen in e.g., *object naming*, i.e., deciding on adequate designation terms for depicted entities (Brown, 1958; Ordonez et al., 2016; Pontillo, 2017; Zarrieß and Schlangen, 2017; Eisape et al., 2020). While naming is indispensable for neural end-to-end approaches, there is no one-to-one relationship between depicted entities and lexical items. For example, in Figure 9, a wide range of possibilities exist for naming the target person, e.g., “person,” “man,” “male,” or “father” (cf. also Silberer et al. 2020a,b). Certain dimensions of naming variation are consistent with the core formulation of the REG task, i.e., variation in *lexical specificity* as a function of distractor context (cf. Graf et al. 2016, among others). However, this is not always the case: For example, Ross and Murphy (1999) suggest that people have alternative organizations (or cross-classifications) for food items that are utilized for different kinds of inferences. A more general way to look at naming variation might be to analyze lexical decisions as reflecting conceptual perspectives in the sense of Gatt and van Deemter (2007), i.e., alternative (and potentially co-extensive) categorizations highlighting the relevance of different sets of semantic aspects.

The general importance of visual context for naming decisions is illustrated in Figure 9: Here, the majority of annotators agree on some variant of *kinship terms* (i.e., “father” and “dad”), instead of using “man,” which would be (a) equally possible given the visual appearance, (b) equally effective in the given situation and (c) more common in the whole dataset. Given the lack of other sources of contextual information, the visual context seems to be crucial in this case. However, due to their focus on negative context, current approaches in visual REG do neither account for the type of contextual information from which kinship relationships are inferred nor for the reasons why this categorization is applied by annotators.

Without further investigation, there are several layers of contextual information that could provide cues for inferring relevant conceptual perspectives. First, in accordance with the importance of object co-occurrence for recognition (Section 5.1) and in line with general assumptions in Gatt and van Deemter (2007), lexical decisions could reflect the relations between distinct objects, i.e., the categorization as *father* is inferred from the co-presence of his suspected wife and children. Alternatively, more global aspects of the scene context could be relevant, such as the scene type (e.g., the *dining room* indicating a family setting). Finally, representations of depicted events and actions might affect naming decisions, e.g., visible scripts, schemata or event roles, the latter of which have been shown to be accessed by humans rapidly and spontaneously upon scene perception (Hafri et al., 2018). Related structures such as semantic *frames* (Fillmore, 1977) could be used to analyze certain dimensions of naming variation, by providing sets of frame-specific semantic roles and associated lexical units in which depicted entities are integrated by the observer. Crucially, all of this information can be regarded as positive context as discussed in Section 3.2, as in all cases, common characteristics of the target and situational context are reflected in lexical decisions. Importantly, here, visual context appears to be embedded in a larger body of semantic information, and reinterpreted based on e.g., world knowledge and personal expectations (van Miltenburg, 2017; Pustejovsky and Krishnaswamy, 2018). For example, in Figure 9, there is no way of *knowing* about the family ties between the depicted persons—however it is collectively *assumed* by all annotators.

Examples like Figure 9 can be regarded as special cases of (lexical) *overspecification*, where naming decisions reflect higher conceptual specificity than required to exclude potential distractors. This raises the question of *why* the annotators include kinship information, even though they are seemingly irrelevant to the primary goal of REG. For this, different hypotheses can be made, including both *addressee-* and *speaker-internal* processes (cf. Koolen et al. 2011, among others): First, some works have described including logically redundant properties in reference as an addressee-oriented behavior, i.e., providing additional information to facilitate the resolution process in complex domains (Paraboni et al., 2006). In this sense, the more specific categorizations inferred from the positive context would indeed be utilized for achieving the primary REG objective, i.e., enabling identification. Alternatively, following Gatt and van Deemter (2007), additional constraints are possible: For example, it is conceivable that the individual people are perceived as members of a common group, resulting in the necessity to satisfy the Conceptual Coherence Constraint, i.e., conceptualize the group members in a coherent way, in order to avoid unintended inferences by the addressee (cf. Section 3.2). As an example for speaker-internal reasons, one might assume priming effects where the context pre-activates lexical fields for naming decisions, similar to scene context pre-activating certain object types (Figure 8). Finally, perspective taking could be motivated by entirely new communicative goals which go beyond enabling identification (cf. Jordan 2000). For example, van Deemter (2016) highlights the general role of *interestingness* in image descriptions, where speakers verbalize information they consider to be *worth saying* because it is surprising, remarkable or standing out in other ways. Translated to reference generation, in Figure 9, a speaker might deem the depiction of a typical family situation to be an important feature of general image, leading to the production of related items for depicted persons. Importantly, this also raises questions about downstream V&L tasks such as Visual Question Answering and Visual Dialogue, which conceptually include REG as a necessary processing step (Sun et al., 2022). In higher-level tasks involving REG, other communicative goals complement the identification of referential targets, potentially leading to additional determinants of object naming and perspective taking.

Overall, positive (visual) context and conceptual perspectives are pressing issues that arise for generating natural expressions with visual inputs. However, fundamental questions remain, for example regarding the representation and processing of larger semantic structures like scene types and frames, integration of world knowledge and prior assumptions, and the pragmatic processes involved in naming decisions.

## 6. Discussion

Research in REG has long been concerned with generating references in visual environments, either by using symbolic representations as a proxy for perceptual object properties or by operating directly on visual representations. Considering the critical role of situational context, its precise implications for the content of the generated expressions have been considered surprisingly little so far—both in symbolic REG, where the focus has long been on aspects such as preference orderings in human descriptions, and in visual REG, where there is some work about visual context (Yu et al., 2016; Li and Jiang, 2018), but with an emphasis on technical issues of integration. It is important to note that the difficulty of accurately describing and formalizing contextual influences is not limited to REG, as even the notion of context itself is notoriously lacking a commonly accepted standard definition in linguistics (Meibauer, 2012). In principle, virtually anything can present a relevant context for producing or understanding linguistic utterances (or, following Spivey and Huette 2016, “there is absolutely nothing that cannot be context,” cf. also Clark 1996). Even if we reduce context to visually perceivable stimuli, tiny aspects of it could trigger complex linguistic reasoning processes, cf. Hunter et al. (2018).

As an initial step toward a deeper understanding of visual context in reference production, we derived a set of *types of context* from symbolic approaches. Applying those categories to the visual REG literature revealed that, despite the increase in modeling complexity, existing approaches primarily focus on distractor context, with some works additionally reflecting notions of salience (e.g., attention mechanisms) or relata (including relations between visible objects). Based on this observation, we highlighted additional ways in which information from the visual context can facilitate reference generation. First, we argued for leveraging scene context to address the inherent uncertainty when detecting and categorizing objects and their properties from perceptual inputs, as shown effective in related fields of research. Second, we illustrated the problem of object naming as part of realizing fully formulated descriptions, and provided examples of sources of positive context indicating adequate conceptual perspectives for naming decisions. Crucially, both types of context integration connect to prior research in related fields but have not been considered for the REG task so far.

However, there are also open questions regarding the types of context reflected in the visual REG literature. For distractor context, various approaches to include contrasting information from the context exist, e.g., visual comparisons, listener components or dedicated decoding strategies. However, for more complex scenes, it is seldom discussed which of the objects in the context should be considered in order to generate distinguishing descriptions. For this, some implicit assumptions can be found in the literature: *Type-based* approaches consider objects as distractors which have types sufficiently similar to the target. For symbolic REG, this is exemplified by the *Visible Objects Algorithm* (Mitchell et al., 2013), where only context objects of the same type trigger comparisons to the target. In a similar vein, Mao et al. (2016); Yu et al. (2016) especially focus on contrasting information from context objects of the same category in their approaches. *Relevancy-based* approaches select distractor objects without relying specifically on their type, as can be seen in work on salience in symbolic REG (Sections 2.1.2, 3.4), but also in attention-based approaches like Kim et al. (2020), where context relevance for visual comparisons is learnt by the model. Finally, *exhaustive* approaches include all co-occurring objects as distractors. Whereas this is the case for the core REG formulation in symbolic REG (Section 2.1.1), it can be also seen in Schüz and Zarrieß (2021)'s approach to visual REG, where all annotated objects co-occurring with the target are considered as distractors during inference. So far, a systematic comparison between these approaches has not been carried out.

For relata and landmarks, the general capability of current systems to generate relational descriptions is far from clear. Whereas some work on visual REG has adapted their models to better capture visible relationships (Li and Jiang, 2018; Tanaka et al., 2019; Kim et al., 2020), to the best of our knowledge, detailed evaluations of REG systems regarding relational expressions are missing in the literature. This includes both evaluations of the prevalence and adequateness of relational expressions in model outputs, and detailed accounts of whether systems actually consider information from the visual input in those cases, given related work from image captioning indicating that generation models often rely on textual information for generating spatial relations (Ghanimifard and Dobnik, 2019). Questions about the capabilities of current systems become even more pressing when different types of relations between objects are considered: Whereas REG has often focused on spatial relations between objects, further kinds of relations can be extracted from visual inputs, including (human) interactions (e.g., “the person kicking the ball,” Nagaraja et al. 2016; Krishna et al. 2018). Importantly, different types of visual relationships might require different ways processing: Whereas spatial relations can be largely deducted from the relative locations of objects, interaction relationships might require more complex processing such as action recognition.

Taken together, by switching to visual inputs and neural modeling paradigms, the REG task has increased in complexity. However, in many regards, underlying concepts are still in line with fundamental work on symbolic REG. For this, the integration of visual context is a case in point: In principle, as shown in this work, information from the situational context can facilitate the generation process and shape the content of generated expressions in many different ways, especially considering the richness of contextual information as provided by natural images. However, in its core principles, the integration of this context remains largely consistent with pioneering approaches in symbolic REG. Therefore, it is necessary to acknowledge the inherent complexity of the visual REG task and to investigate the implicit processing steps and goals in more detail. Some of the general directions of future work in REG might include: (a) more detailed evaluations of existing systems, in order to get a more thorough picture of the linguistic abilities of the systems and the contextual information they integrate; (b) extending REG datasets such as RefCOCO with more comprehensive visual and linguistic annotations, to allow for corpus-based analyses of different types of context; (c) the identification and description of the more implicit challenges of the REG task, such as object recognition and naming, to establish and strengthen connections to other disciplines concerned with related tasks; (d) a more focused investigation and operationalization of pragmatic goals in REG that go beyond identification (such as ease of comprehension and conceptual coherence), in order to enable the evaluation of communicative success in those regards; and (e) exploring REG as a conceptual component of higher-level V&L tasks, where the primary goal of REG (enabling identification) is embedded in further, task-specific objectives.

For visual REG modeling, this article has shown that context objects can play different roles in the generation of referring expressions. Future research should therefore explore methods to better exploit this variability in generation models. For this, dynamic context representations that allow generation models to flexibly capture different types of relationships between referential targets and surrounding objects are an important step, see Li and Jiang (2018); Sun et al. (2022) for existing approaches using RNNs or Transformer cross-attention. Complementary research should explore the representation of more global features of visual context: As with symbolic REG, research in visual REG has focused primarily on situational context in the form of co-occurring objects. However, as described in Sections 5.1, 5.2, information about e.g., scene types or depicted actions is more global in nature. So far, it is unclear to what extent this type of information is captured in existing contextual representations and whether it is reflected in the outputs of neural generation models.

## 7. Conclusion

For Referring Expression Generation, information from the situational context is crucial, as it determines whether a given expression unambiguously identifies a referent in a given situation. However, context is notoriously hard to capture, as it lacks commonly accepted definitions and virtually anything can present a relevant context in situated communication. In REG, characterizing contextual influences has further been complicated in recent years, due to an increasing shift to multimodal settings and neural generation models, operating on raw visual instead of symbolic information.

As an initial step toward a deeper understanding of visual context in REG, we utilized the cross-paradigm formulation of this task, and derived a set of *types of context* based on the different ways in which situational context affects content determination in symbolic REG. After this, we turned our view on visual REG, to see whether similar kinds of contextual integration can be found in existing approaches. After highlighting some limitations in this regard, we discussed possible ways in which visual context can be leveraged to address some of the challenges brought in by the switch to raw perceptual information in visual REG.

For future research, we see great potential in investigating how information from the visual context can be utilized to address the implicit challenges posed by the increased complexity of the visual REG task.

## Author contributions

SS and SZ contributed to conception. SS conducted the investigation and wrote the draft of the manuscript with input from all authors. All authors contributed to manuscript revision, read, and approved the submitted version.
